# Estimation of the Land Surface Temperature over the Tibetan Plateau by Using Chinese FY-2C Geostationary Satellite Data

**DOI:** 10.3390/s18020376

**Published:** 2018-01-28

**Authors:** Yuanyuan Hu, Lei Zhong, Yaoming Ma, Mijun Zou, Kepiao Xu, Ziyu Huang, Lu Feng

**Affiliations:** 1 Laboratory for Atmospheric Observation and Climate Environment Research, School of Earth and Space Sciences, University of Science and Technology of China, Hefei 230026, China; hyy1993@mail.ustc.edu.cn (Y.H.); zoumj@mail.ustc.edu.cn (M.Z.); xukp@mail.ustc.edu.cn (K.X.); hzy17@mail.ustc.edu.cn (Z.H.); 2 Key Laboratory of Tibetan Environment Changes and Land Surface Processes, Institute of Tibetan Plateau Research, the Chinese Academy of Sciences, Beijing 100101, China; ymma@itpcas.ac.cn; 3 CAS Center for Excellence in Tibetan Plateau Earth Sciences, Beijing 100101, China; 4 University of Chinese Academy of Sciences, Beijing 100049, China; 5 Institute of Tropical and Marine Meteorology, China Meteorological Administration, Guangzhou 510080, China; fengl@grmc.gov.cn

**Keywords:** FengYun 2C, split window algorithm, land surface temperature, the Tibetan Plateau

## Abstract

During the process of land–atmosphere interaction, one of the essential parameters is the land surface temperature (LST). The LST has high temporal variability, especially in its diurnal cycle, which cannot be acquired by polar-orbiting satellites. Therefore, it is of great practical significance to retrieve LST data using geostationary satellites. According to the data of FengYun 2C (FY-2C) satellite and the measurements from the Enhanced Observing Period (CEOP) of the Asia–Australia Monsoon Project (CAMP) on the Tibetan Plateau (CAMP/Tibet), a regression approach was utilized in this research to optimize the split window algorithm (SWA). The thermal infrared data obtained by the Chinese geostationary satellite FY-2C over the Tibetan Plateau (TP) was used to estimate the hourly LST time series. To decrease the effects of cloud, the 10-day composite hourly LST data were obtained through the approach of maximal value composite (MVC). The derived LST was used to compare with the product of MODIS LST and was also validated by the field observation. The results show that the LST retrieved through the optimized SWA and in situ data has a better consistency (with correlation coefficient (R), mean absolute error (MAE), mean bias (MB), and root mean square error (RMSE) values of 0.987, 1.91 K, 0.83 K and 2.26 K, respectively) than that derived from Becker and Li’s SWA and MODIS LST product, which means that the modified SWA can be applied to achieve plateau-scale LST. The diurnal variation of the LST and the hourly time series of the LST over the Tibetan Plateau were also obtained. The diurnal range of LST was found to be clearly affected by the influence of the thawing and freezing process of soil and the summer monsoon evolution. The comparison between the seasonal and diurnal variations of LST at four typical underlying surfaces over the TP indicate that the variation of LST is closely connected with the underlying surface types as well. The diurnal variation of LST is the smallest at the water (5.12 K), second at the snow and ice (5.45 K), third at the grasslands (19.82 K) and largest at the barren or sparsely vegetated (22.83 K).

## 1. Introduction

During the process of land–atmosphere interaction, the land surface temperature (LST) is a pivotal parameter for characterizing energy as well as the mass exchange between the atmosphere and the ground surface [1]. It is also crucial to the study of the surface energy balance [2,3,4,5] and is extensively needed in a variety of fields, especially in meteorology, geology, hydrology, and ecology, among others [6,7,8,9,10]. Reputed as the world’s highest plateau and the “Roof of the World”, an average elevation of the Tibetan Plateau (TP) is approximately 4000 m. Because of its thermodynamic and dynamic effects, the TP is vitally important to the atmospheric circulation and climate change in Asia and even the Northern Hemisphere [11]. According to prior research, the thermal effects of the TP greatly influence the monsoon outbreak and atmospheric circulation [12,13,14,15,16]. Under the global warm climate, it is vital to investigate the heating source changes in the TP; meanwhile, the LST is one of the heating-source indicators [17]. As such, it is essential to accurately gain the spatio-temporal distribution of the LST in the TP area.

However, the traditional way of LST ground measurement is conducted at the point scale. It is insufficient to meet the current regional-scale application requirements [18,19]. Fortunately, satellite remote sensing technology can partially make up for these deficiencies. The thermal infrared data and the remote sensing approaches are utilized to describe LST on a regional or even global scale [20,21,22,23]. According to the thermal bands involved, the LST retrieval methods could be cursorily divided into three categories: single channel method, multiangle method and multichannel method [23]. Recent research has developed new physically based schemes, which retrieve simultaneously LST and land surface emissivity (LSE) with high accuracy [24,25,26,27,28,29]. Based on static physical schemes and observational data from multiple time steps, Li et al. [24] and Li et al. [25] derive the LST and LSE simultaneously. Masiello et al. [26], Masiello and Serio [27], and Masiello et al. [28] utilized the Kalman filter (KF) method, which is a dynamical physical-based scheme and novel approach to directly retrieve LST and three channels’ LSEs. The accuracy of the retrieval result is about ±0.2 °C and ±0.005, respectively. Rozenstein et al. [29] used the KF approach to study the diurnal LSE dynamics in a coastal desert area, which further demonstrated the reliability of the KF method. However, the traditional KF algorithm is only applicable to the linear Gaussian system. In addition, these simultaneous methods are more complex and only applicable to experimental setups, which may limit the wide use of these simultaneous approaches [30]. Operationally, as one of the most widely used and the most fully developed multichannel algorithms, the split window algorithm (SWA) available to derive the LST precisely without atmospheric vertical profile information. At present, more than twenty SWAs have been published. Based on two different polar-orbiting satellites data (Advanced Very High Resolution Radiometer (AVHRR) and the Moderate Resolution Imaging Spectroradiometer (MODIS)), Sobrino et al. [31,32] utilized a novel SWA to retrieve the LST with high precision. On the basis of simplifying the radiation transfer equation, Qin et al. [33] proposed a SWA that involves only emissivity and transmittance parameters and the accuracy of retrieval LST is below 2 K. Besides the AVHRR [18,19,31,33,34] and MODIS [32,35,36], the SWA has been succeeding in the application for estimating the LST from instruments onboard other polar-orbiting satellites, including the Landsat-8 Thermal Infrared Sensor (TIRS) [37,38], the Advanced Spaceborne Thermal Emission and Reflection Radiometer (ASTER) [39], etc. Since the LST has a high temporal variability that is especially large in a diurnal cycle [10], it is often inadequate to use the LST retrieved by polar-orbiting satellites, which has low temporal resolution, for many applications such as numerical weather forecasting and ecosystem process modeling.

Meanwhile, many geostationary meteorological satellites have been launched in space successfully and hourly remote sensing data have gained increasing attention. The geostationary meteorological satellite has a high time resolution [40], and the diurnal variation of various parameters of the surface can be captured. Therefore, the hourly LST data can be obtained using the geostationary meteorological satellite data. Tang et al. [41] estimated the LST for cloud-free skies around Beijing in China from data from FengYun-2C (FY-2C), which are the first operated geostationary meteorological satellites of China. However, the results have not yet been evaluated due to lacking of in-situ observations. Ouyang and Li [42] modified a general SWA and a cross-calibration method for the FY-2C/the Stretched Visible and Infrared Spin Scan Radiometer (SVISSR) satellite data and calculated the LST in the area of Heihe River Basin. The retrieval LST was validated after the aggregated Advanced Along-Track Scanning Radiometer (AATSR) derived the LST with in situ observations. The SVISSR and aggregated AATSR gave comparable results (within 4 K). However, there was an overestimation of approximately 3 K for the AATSR LST product compared to the ground measurements, and the LST was retrieved only for two days (7 and 10 December 2007). Jiang and Liu [43] improved the SWA using the moderate spectral resolution atmospheric algorithms and computer model (MODTRAN) and retrieved the LST from the FY-2C/D/E (located above the equator at longitude 105° E, 86.5° E and 123.5° E, respectively) measurements. The retrieval of the LST was cross-validated with the MODIS products (the total errors of the FY-2C/D/E are 0.3±1.6 K, 0.3±1.9 K, and 1.0±1.8 K, respectively). However, the data from only two time spans (September 2007 for FY-2C/D/SVISSR and May 2010 for FY-2E/SVISSR) were chosen to calculate the LST. Oku and Ishikawa [44] calculated the hourly LST from the Geostationary Meteorological Satellite Visible/Infrared Spin-Scan Radiometer (GMS VISSR) data over the TP in 1998. The correlation coefficient is approximately 0.8 between the calculated LST and in situ observations, and there is a large root mean square error (RMSE) of nearly 10 K.

There are actually some published SWAs to calculate the LST. However, if the coefficients in SWAs are not optimized, the existing SWAs are not suitable for estimating the LST over a certain region, especially for the most challenging area of the TP. For the time being, there is also a lack of LST products with high accuracy and temporal resolution. Thus, this paper aims to establish a SWA suitable for the retrieval time series of LST with resolution and high accuracy over the TP. The spatio-temporal LST variation patterns and its relationship with environmental factors will also be identified. The structure of the article is as below. The introduction of the data and study area is shown in Section 2. In Section 3, the methodology adopted in this study is introduced. The validation results of LST derived from modified SWA, the spatio-temporal LST variation characteristics and its relationship with the underlying surface types are presented in Section 4. The last part provides the conclusions, including concluding remarks and discussion.

## 2. Data and Study Area

### 2.1. Data

The data used in this paper mainly include satellite data (FY-2C/SVISSR, Terra/MODIS), reanalysis product (the Climate Forecast System Reanalysis (CFSR) project of the National Centers for Environmental Prediction (NCEP), the Modern-Era Retrospective analysis for Research and Applications, Version 2 (MERRA-2)), GPS water vapor content (WVC) data and in situ observations. The detailed introductions of above datasets are as follows. Launched on 19 October 2004 and developed by the Chinese Academy of Space Technology (CAST) as well as the Shanghai Academy of Space Flight Technology (SAST), the FY-2C, as the fourth satellite of the FY series and the first meteorological satellite operated by China, became completely operational in 2006. When the FY-2C is above the equator at longitude 105° E with the distance of 35,800 km away, with a half-hour acquisition interval in the flood season, the area ranges from 45° E to 165° E longitude and from 60° S to 60° N latitude per hour. SVISSR is the major sensor onboard the FY-2C, consisting of four infrared channels (FIR1: 10.3–11.3 μm, FIR2: 11.5–12.5 μm, FIR3: 6.3–7.6 μm, MIR: 3.5–4.0 μm) and one visible channel (VIS: 0.55–0.90 μm) [42]. Table 1 shows the characteristics of FY-2C/SVISSR channels. The FY-2C HDF data format, a 5-km normalized projection full disc image (NOM), was used and downloaded from the FY Satellite Data Center (http://satellite.nsmc.org.cn/PortalSite/Default.aspx). The data selected in this study are the daytime data (8:00 a.m. to 7:00 p.m. Beijing Standard Time (BST)) of the year 2008.

As the major instrument aboard the satellites of Aqua and Terra, the Moderate Resolution Imaging Spectroradiometer (MODIS) consists of 36 spectral channels with the wavelength between 14.4 µm and 0.4 µm, of which the 29–36 channels are the thermal infrared ones utilized to supervise the variations of heat at the surface of Earth [45,46]. The temperature and emissivity values from the MOD11C1 V41 product, configured on a 0.05° latitude/longitude climate modeling grid (CMG), are obtained by reprojection and the average of the values in the daily MODIS LST and emissivity (LST/E) product (MOD11B1) is calculated at 5 km equal area grids in the sinusoidal projection [47]. In this work, the daily and monthly MODIS/Terra LST/E products (MOD11C1 and MOD11C3 V41 product) in 2008 were downloaded from MODIS data web (https://modis.gsfc.nasa.gov/data/).

The NCEP CFSR over the 31-year period from 1979 to 2009 was initially completed in January 2010. The CFSR is the latest set of the NCEP global, coupled reanalysis data that uses the interactive sea ice model and three-dimensional variational assimilation technology. The NCEP CFSR project has hourly precipitable water data with Gaussian projection. The unit of the NCEP CFSR WVC product is $\mathrm{kg}/m^{2}$. The latest atmospheric study in the modern era of satellite produced by NASA’s Global Modeling and Assimilation Office (GMAO) is version 2 of MERRA. The products of MERRA-2 can be accessed online via the NASA Goddard Earth Sciences Data Information Services Center (GES DISC) [48]. The MERRA-2 products have hourly precipitable water data with latitude/longitude projection with spatial resolutions of 0.5° × 0.667°. The unit of the MERRA-2 WVC product is same as the NCEP CFSR reanalysis data.

**Figure 1:**
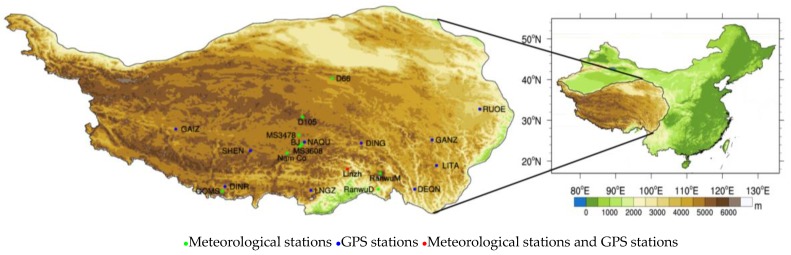
Location of the Tibetan Plateau. Circles of different colours represent the meteorological and GPS stations scattered in the Tibetan Plateau.

The in situ observations are from the Enhanced Observing Period (CEOP) of the Asia-Australia Monsoon Project (CAMP) on the Tibetan Plateau (CAMP/Tibet). Eight stations were selected, namely, BJ, D66, D105, MS3478, MS3608, Nam Co, Linzhi and QOMS (Qomolangma Station for Atmospheric Environmental Observation and Research, Chinese Academy of Sciences (CAS)) (Table 2 and Figure 1). Using the Equation (1), the in situ LST was calculated from the downward and upward longwave radiation flux, which were observed at stations by Kipp and Zonen CNR1 net radiometers [39]:

(1) $$T{\;}_{g}={\left(\frac{R_{lw}{}^{↑}-\left(1-\varepsilon_{s}\right)R_{lw}{}^{↓}}{\varepsilon_{s}\sigma }\right)}^{1/4},$$

in which $T_{g}$ is the LST; $R_{lw}{}^{↓}$ and $R_{lw}{}^{↑}$ are the downwelling and upwelling longwave radiation flux, respectively; σ is the Stefan–Boltzman constant ($\sigma =5.67\times 10^{-8}\;W\cdot m^{-2}\cdot K^{-4}$); and $\;\varepsilon_{s}$ is the surface emissivity that can be calculated form MODIS narrowband emissivity (MOD11C3 product) by using a linear equation below [49,50]:

(2) $$\varepsilon_{s}=0.2122\varepsilon_{29}+0.3859\varepsilon_{31}+0.4029\varepsilon_{32},$$

where $\varepsilon_{32}$, $\varepsilon_{31}$ and $\varepsilon_{29}$ are the narrowband emissivities of the MODIS bands 32, 31 and 29, respectively. In addition to the eight stations above, the observation data of RanwuM and RanwuD (National Earth System Science Data Sharing Infrastructure, National Science & Technology Infrastructure of China (http://www.geodata.cn)), which was used to observe the South-Eastern Tibet Ranwu lake water temperature, have been used in this paper.

The atmosphere of the TP was monitored with the use of the project of the Japan International Cooperation Agency (JICA). We used the GPS atmospheric precipitable water vapor (PWV) measurement data (unit: cm) from the JICA to validate the NCEP CFSR reanalysis data in this work. PWV refers to the amount of precipitation that can be produced when the WVC in the vertical column from the ground to the top of the atmosphere condenses and falls to the ground [51]. According to its physical definition, the GPS original unit cm can be converted into $g/{\mathrm{cm}}^{2}$ by multiplying the PWV data with the density of liquid water [52]. Eleven GPS stations were selected, namely, GAIZ (Gaize), GANZ (Ganze), LITA (Litang), LNGZ (Longzi), NAQU (Naqu), RUOE (Ruoergai), SHEN (Shenzha), DEQN (Deqin), DING (Dingqing), DINR (Dingri) and Linzhi. Table 2 and Figure 1 show the general information of the chosen GPS stations.

### 2.2. Study Area

The TP, often called the “Third Pole of the Earth”, and “Roof of the Word”, is the highest plateau in the world and is located at 26° N–40° N, 70° E–105° E. As China’s largest plateau, TP covers an area of 2.5 million square kilometers. The topography of the study area as well as the locations of GPS stations and the meteorological stations are shown in Figure 1. For a long time, there has been a lack of observations of surface hydro-meteorological parameters due to the harsh climate conditions and the unique geographical environment over the TP.

## 3. Methodology

**Figure 2:**
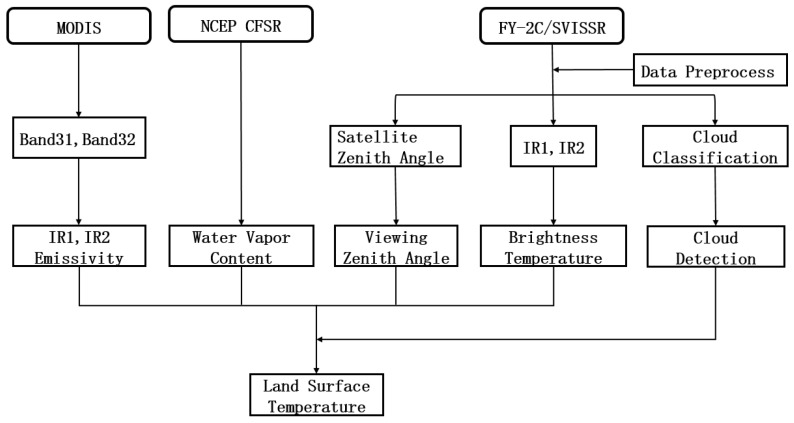
Flowchart of the FY-2C LST retrieval approach.

The key parameters for the estimation of the LST by the SWA are the ground surface emissivity, atmospheric WVC and thermal brightness temperature from the remote sensing thermal infrared channel. Figure 2 gives the flowchart of the LST estimation method applied to the FY-2C data.

### 3.1. Determination of Surface Emissivity

Some algorithms have been proposed to estimate the LSE, including the method based on the land surface classification [53], the day/night method [47], the baseline fit method [43,54] and the Kalman filter physical method [27,28]. Due to the lack of the near-infrared channel of the FY-2C/SVISSR instrument, the present approaches cannot be directly used to the retrieval of LSE from the measurements of FY-2C/SVISSR [43]. Since previous research has validated the daily product of MODIS/Terra LST/E V41 [55], the LSEs of channels 1 and 2 of the FY-2C can be replaced by the LSEs of bands 31 and 32 of the MOD11C1 V41 product [41]. Although the underlying surface is very complicated over the TP [56], from the view of the MODIS 1 km spatial resolution, there are roughly four land-cover types: bare soil, vegetation, snow/ice and water [36]. Therefore, thermal band emissivities for the above underlying surface types were determined by following methods [41], which are adapted to the above four land cover types with high accuracy (the value of RMSEs within 0.002):

(3) $$\varepsilon_{FIR1}=-0.0611+1.0614\varepsilon_{31},$$

(4) $$\varepsilon_{FIR2}=-0.0210+1.0199\varepsilon_{32}.$$

where $\varepsilon_{FIR1}$ and $\varepsilon_{FIR2}$ are the LSEs of channels FIR1 and FIR2 of the SVISSR, while $\varepsilon_{32}$ and $\varepsilon_{31}$ are the LSEs of the MODIS bands 32 and 31, respectively.

### 3.2. Validation and Comparison of the Water Vapor Content

**Figure 3:**
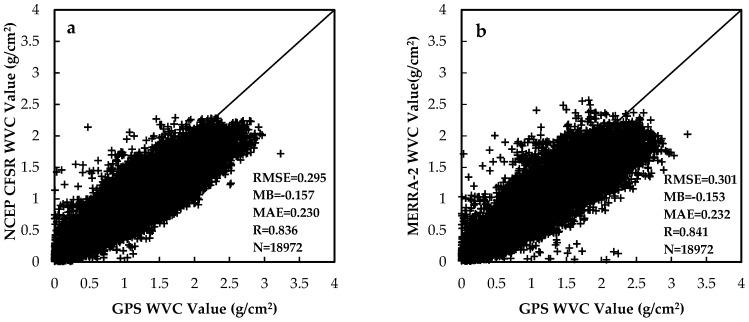
Comparison between the water vapor content from NCEP CFSR reanalysis (**a**); MERRA-2 reanalysis data (**b**) and GPS measurements at 11 stations.

The WVC has large spatial and temporal variations and is difficult to obtain. Although there are some radiosonde data over the TP, the coverage is only at local scale. There are some water vapor reanalysis data, which can provide regional scale water vapor information. However, most of them have lower temporal resolution. Both the NCEP CFSR and MERRA-2 water vapor reanalysis data have a fine temporal resolution of one hour. Thus, they were chosen and validated with the GPS measurements (as the WVC truth data) to find a WVC reanalysis dataset with higher accuracy, which also will better meet the requirements of the FY-2C LST retrieval. To mitigate temporal-spatial matching errors, the NCEP CFSR reanalysis data were re-projected to the WGS-84 projection with space resolution of 0.315° × 0.3125°. Then, the nearest neighbor interpolation method was utilized to resample the image data. Finally, the two WVC data were calculated with a space resolution of 0.05°. To compare the NCEP CFSR and MERRA-2 reanalysis data, the units are standardized to $g/{\mathrm{cm}}^{2}$. Figure 3 shows the comparison between the WVC from NCEP CFSR reanalysis (a); MERRA-2 reanalysis data (b) and GPS measurements at 11 stations. The black solid line is the 1:1 line, and N is the number of data used for cross-validation. The comparison between the reanalysis data of NCEP CFSR and the in situ observations presents better agreement, with correlation coefficient (R), mean absolute error (MAE), mean bias (MB) and RMSE values of 0.836, 0.230 $g/{\mathrm{cm}}^{2}$, −0.157 $g/{\mathrm{cm}}^{2}$ and 0.295 $g/{\mathrm{cm}}^{2}$, respectively. Though there are also good agreement between the GPS measurements and the MERRA-2 WVC product with MB (−0.153 $g/{\mathrm{cm}}^{2}$) and R (0.841), the MERRA-2 data has higher RMSE (0.301 $g/{\mathrm{cm}}^{2}$) and MAE (0.237 $g/{\mathrm{cm}}^{2}$) than the NCEP CFSR product. The statistics of above two reanalysis WVC data versus GPS measurements by stations are listed in Table 3. The accuracy of the two reanalysis WVC products over the TP is very close. However, the NCEP CFSR WVC product is slightly better than that of MERRA-2 for most cases. Thus, the WVC data from the NCEP CFSR were used as inputs for the SWA to retrieve the LST over the TP in this study.

### 3.3. Cloud Detection

Because of the TP’s heterogeneous underlying surfaces, large coverage and complex topography, LST estimation is not an easy task over this challenging area. Convective clouds develop strongly over the TP, especially in summer afternoons. Instead of the LST, the cloud-top temperature is detected by the satellite instruments over cloudy areas. Therefore, a cloud detection should be carried out first. The FY-2C cloud classification information [57] is listed in Table 4. The cloud classification data value of 0 or 1 indicates that the pixel is under the clear-sky condition. Otherwise, this pixel is contaminated by clouds. Based on the FY-2C cloud classification data, each pixel will be identified under cloud effects or not, so the FY-2C images could be classified into three categories of cloud, land and water, respectively.

### 3.4. Split Window Algorithm

As widely known, McMillin [58] first used the SWA to retrieve the temperature of sea surface. Since the late 1980s, different SWAs have been proposed and utilized to estimate LST. According to the characteristics of the SVISSR sensor onboard the FY-2C, the SWA modified by Becker and Li (1995, hereinafter BL95) [59] is utilized, and the LST can be derived as:

(5) $$T_{s}=a_{0}+a_{1}w+\left[a_{2}+\left(a_{3}+a_{4}wcos\theta \right)\left(1-\varepsilon \right)-\left(a_{5}+a_{6}w\right)\Delta \varepsilon \right]\frac{T_{i}+T_{j}}{2}+\;\left[a_{7}+a_{8}w+\left(a_{9}+a_{10}w\right)\left(1-\varepsilon \right)-\left(a_{11}+a_{12}w\right)\Delta \varepsilon \right]\frac{T_{i}-T_{j}}{2},$$

with $\varepsilon =\left(\varepsilon_{i}+\varepsilon_{j}\right)/2$ and $\Delta \varepsilon =\varepsilon_{i}-\varepsilon_{j}$, where $T_{i}$ and $T_{j}$ are the brightness temperature (BT) surveyed in channel i and j centered at 11.0 μm and 12.0 μm, respectively; $\varepsilon_{i}$ and $\varepsilon_{j}$ are the land surface emissivities (LSEs) of channel i and channel j, respectively; ε is the average emissivity; Δε is the emissivity difference of channel i and j; w is the atmosphere WVC; θ is the viewing zenith angle (VZA); and $a_{0}$–$a_{12}$ are the model coefficients. The FY-2C cloud classification data was used to select the satellite data (FY-2C, MODIS), the NCEP CFSR reanalysis data and the field measurements under clear-sky conditions. To achieve a more proper algorithm to retrieve LST over the TP area, a regression method based on in situ LST measurements from seven stations (D66, D105, MS3608, MS3478, Nam Co, Linzhi and QOMS) were used to determine the coefficients in Equation (5). Since both the LSEs and WVC have large temporal heterogeneity, a monthly SWA coefficients look-up table has been established. Due to the lack of water temperature observations in 2008, the in situ water temperature data in 2009 was used to train the model to get the coefficients, which is suitable for water bodies. The data from the site RanwuD was used for calculating the regression coefficients, while the data of another site RanwuM was used for the validation.

The model coefficients ($a_{0}$–$a_{12}$) were listed in Table 5.

After achieving the model coefficients, a sensitivity analysis was run to analyze how significant the effect of the WVC error on the retrieval of LST in the improved SWA. The uncertainties of WVC were divided into six sub-ranges with an overlap of 0.1 $g/{\mathrm{cm}}^{2}$. Table 6 shows the statistical results of WVC sensitivity analysis. According to the statistical coefficients, when the uncertainties of WVC are set to $\pm 0.4\;g/{\mathrm{cm}}^{2}$, $\pm 0.3\;g/{\mathrm{cm}}^{2}$, $\pm 0.2\;g/{\mathrm{cm}}^{2}$ and $\pm 0.1\;g/{\mathrm{cm}}^{2}$, the RMSEs between the actual and retrieved LST are 6.30 K, 4.72 K, 3.14 K and 1.57 K, respectively. The MBs are ±5.02 K, ±3.77 K,  ±2.51 K and ±1.26 K, respectively. The MAEs are 5.39 K, 4.04 K, 2.69 K and 1.35 K, respectively. Therefore, in the retrieval of LST, the accuracy of WVC cannot be ignored.

## 4. Results and Discussion

**Figure 4:**
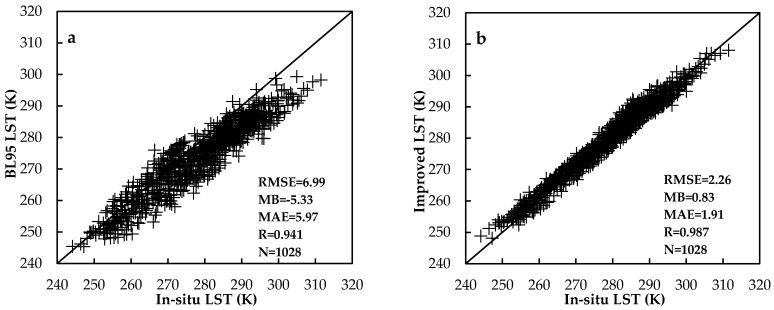
Validation between the retrieved LST from BL95; (**a**) the improved SWA; (**b**) against the field measurements (one-year data).

A total number of eight stations with LST measurements were available in this study, seven of which were used to build the SWA while one independent station was used to do the cross validation. Figure 4 demonstrates the validation between the retrieved LST from BL95 (a) (using the BL95 regression coefficients [59]), the improved SWA; and (b) against the field measurements (one-year data). As shown in Figure 4, the improved SWA has lower RMSE (2.26 K), MB (0.83 K), MAE (1.91 K), and higher R (0.987) than BL95 (with RMSE, MB, MAE and R values of 6.99 K, −5.33 K, 5.97 K and 0.941, respectively), which means that the improved SWA could provide a better estimation of LST than the BL95. According to the statistical results above, it is obvious that there is no universal SWA. Especially for the area of the TP, it is necessary to modify a SWA to achieve LST with high accuracy according to local surface and atmospheric conditions.

**Figure 5:**
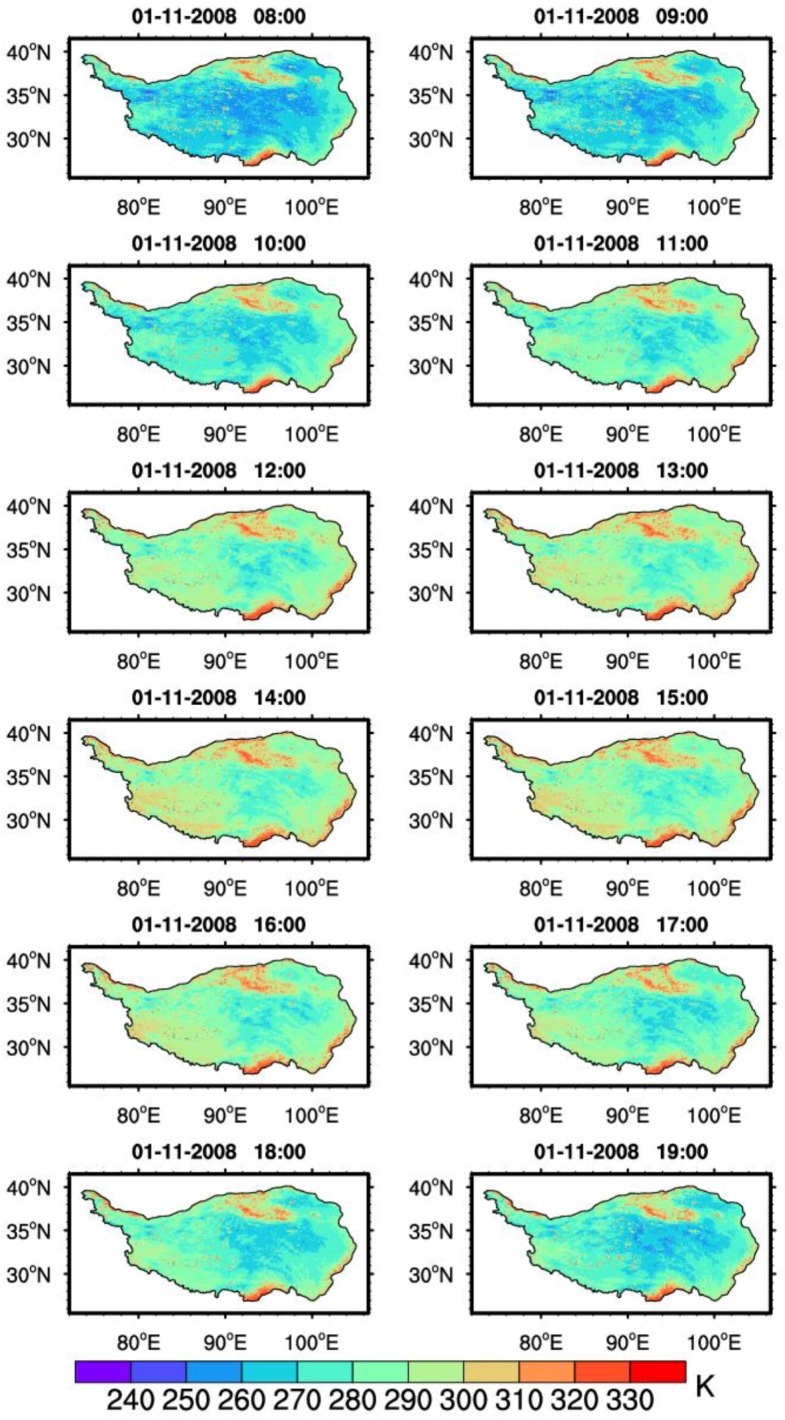
Spatio-temporal distribution of the retrieved LST from 8:00 a.m. to 7:00 p.m. BST, 1 November 2008.

Using the improved SWA, the LST in 2008 was calculated over the TP. To reduce the cloud impact, the MVC method [60] was applied for each pixel in the image. The composite data are produced for every 10-day interval from the derived LST. Figure 5 demonstrates the spatio-temporal distribution of the 10-day composite hourly LST from 8:00 a.m. to 7:00 p.m. BST on 1 November 2008. It is easy to see that the diurnal variation of the LST is distinct over the TP area. From 8:00 a.m. to 7:00 p.m. (BST), the LST increases by more than 30 K in the western parts of the TP, while there is only a slight increase in the LST in the eastern and central area of the Plateau.

**Figure 6:**
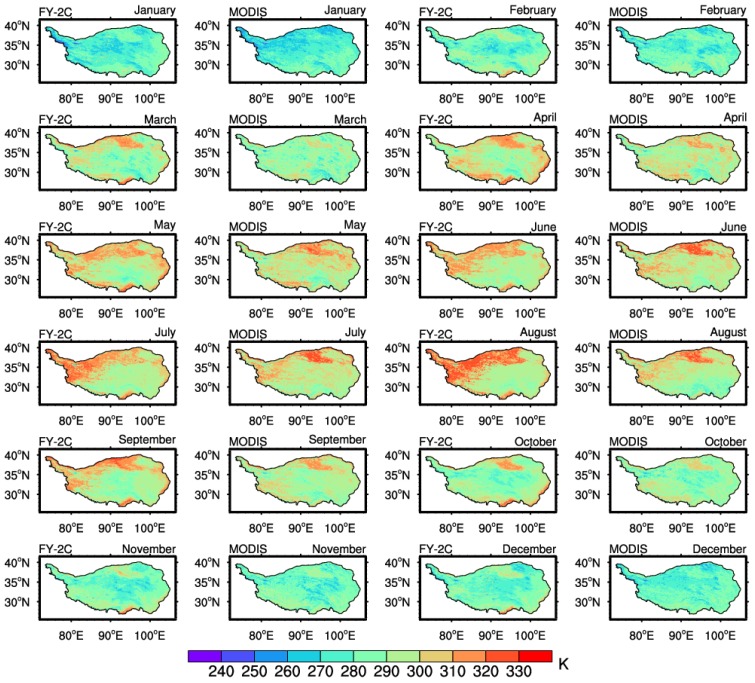
Spatio-temporal distribution of the FY-2C LST and MODIS product.

**Figure 7:**
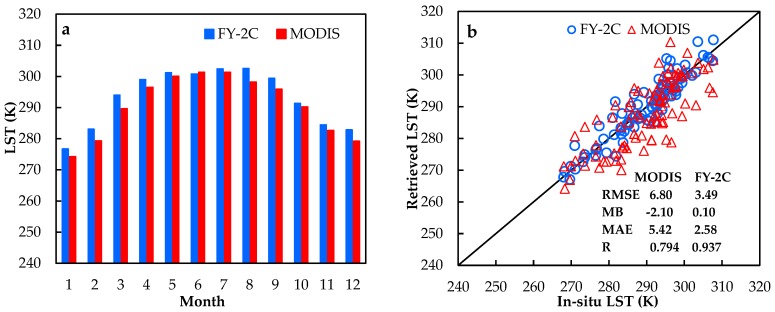
Comparison between derived MODIS LST product and FY-2C LST (one-year data). (**a**) the seasonal variations of the average LST derived from MODIS and FY-2C over the entire Tibetan Plateau; (**b**) the validation of the MODIS and FY-2C results against the in situ measurements.

To further validate the improved SWA for the FY-2C, the MODIS LST products were chosen for comparison. As is well known, the transit time of the Terra satellite is at approximately 10:30 a.m. (local time). Therefore, for contrasting to the MODIS LST product, the average value of the retrieved LST at two time points closest to the transit time was calculated. The detailed spatio-temporal distribution is shown in Figure 6. It can be observed that the LST of these two datasets has a similar spatial distribution. The monthly mean values of the LST show clear seasonal variation. The southern and western parts of the TP and the Qaidam Basin region can be clearly identified as high value centers, and the area of the Kunlun Mountains has relatively low LST due to its high altitude. Figure 7a shows the histograms of the monthly average LST values over the TP from the FY-2C retrieval and MODIS product. The MODIS LST product is usually lower than the derived LST. Subsequently, the in situ observations were utilized to evaluate the retrieved LST and MODIS product. The RMSE, MB, MAE and R of the MODIS product are 6.80 K, −2.10 K, 5.42 K, and 0.794, respectively, while those of the derived FY-2C LST are 3.49 K, 0.10 K, 2.58 K and 0.937. The derived FY-2C LST using this modified SWA is more accurate than the MODIS product over the TP area.

**Figure 8:**
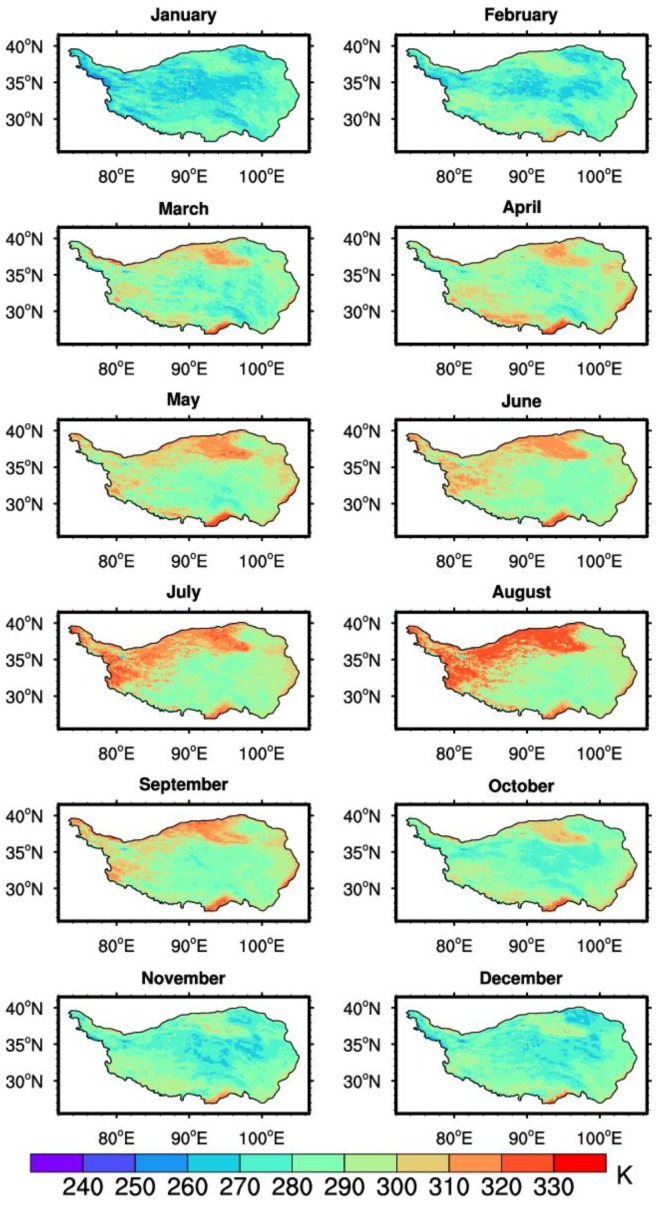
Spatio-temporal distributions of the monthly mean of the daily LST maximum in 2008.

**Figure 9:**
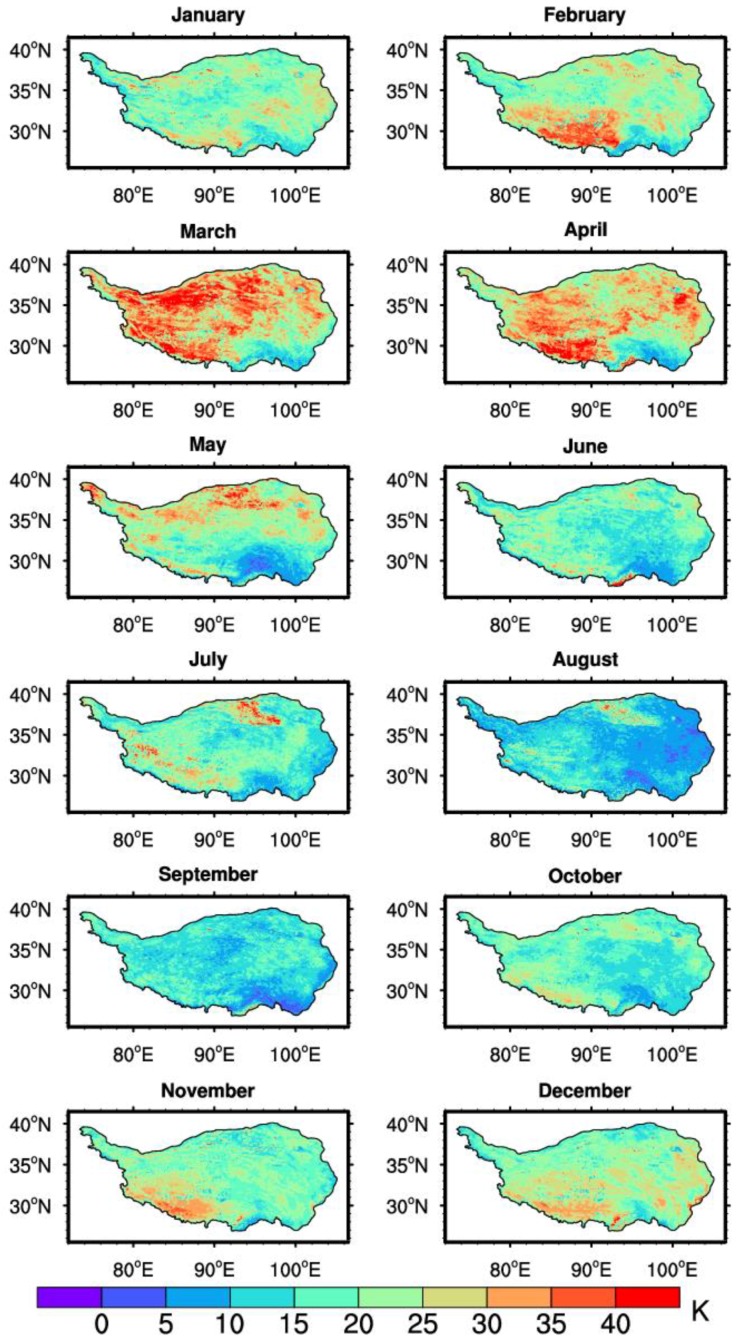
Spatio-temporal distributions of the monthly mean diurnal range of the LST in 2008.

The monthly mean of the daily maximum and minimum LSTs were further derived to study the LST variations in different regions of the TP. Figure 8 demonstrates the spatio-temporal distribution of the monthly mean of the daily maximum LST in 2008. The daily maximum LST in the central part was lower than that in other parts of the TP. From April to October, there were clearly greater LST centers of daily maximum LST in the southern and western parts of the TP and the Qaidam Basin area. This may be due to the difference of the underlying surface conditions. The western part of the TP and Qaidam Basin area (where a desert is located) are relatively dry, and radiative cooling during night is expected to be much stronger there than that in the east. Besides the surface of Qaidam Basin area and the western plateau are much easier to heat up and cool down. Subsequently, the difference between the monthly mean daily LST maximum and minimum was computed. The spatio-temporal distributions of the monthly mean diurnal range of the LST are shown in Figure 9. Especially in March, the diurnal variation of the LST is higher than 25 K in both the southwest and northeast part of the TP during the period between January to April and from October to December. Compared to the months mentioned above, the diurnal range of the LST in other months is relatively low. The summer monsoon over the TP usually begins in May and persists until late September with a certain amount of rainfall during the monsoon period [61]. In addition, from mid-May, the frozen soil enters a complete thawing stage. Due to an increase in precipitation in the mid-monsoon period and soil thawing, the soil heat capacity increases, which leads to a drop in the diurnal range of the LST. Therefore, the rainy season in 2008 and soil thawing could explain why the diurnal range of the LST was less obvious from May to September.

**Figure 10:**
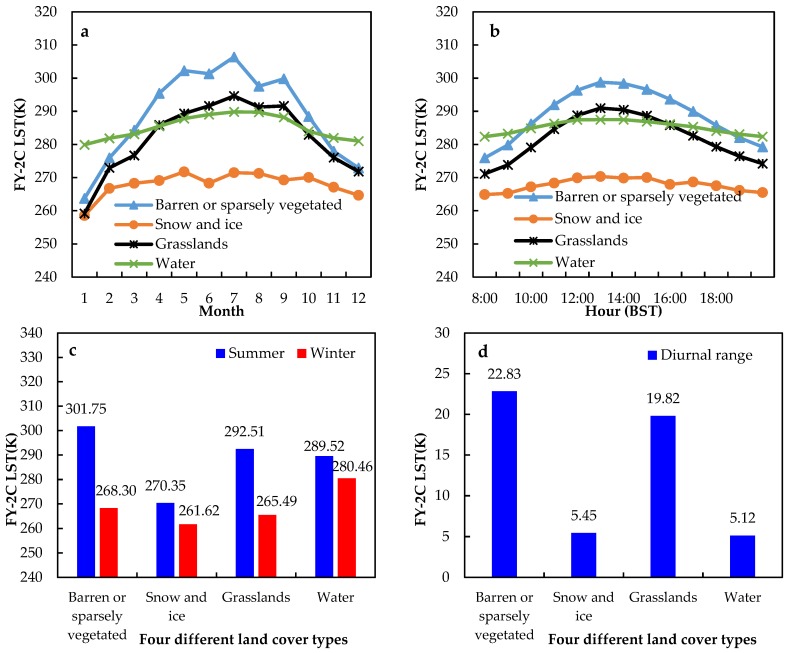
(**a**) the seasonal variations of the monthly average LST for these different land cover; (**b**) the diurnal variations of the LST of those different underlying surfaces; (**c**) the average LST of four different underlying surfaces in summer and winter; (**d**) the diurnal LST range of four different land cover types.

Four typical underlying surface types were selected over the TP to conduct further research on the effects of the different land cover on LST variations. Instead of randomly selecting four underlying surfaces, all pixels with the same land cover have been extracted and averaged. According to Chen et al. [62], when the albedo is greater than 0.47, the underlying surface type can be regarded as snow and ice. The other three underlying surface types were identified by using a MODIS underlying surface type product (MOD12C1). Figure 10a shows the seasonal variations of the monthly average LST for these different land covers. It is easy to see that the seasonal variation of monthly average LST of four different underlying surface types has the same single peak variation pattern, and all of them reached their maximum in June, except for the barren or sparsely vegetated. The average LST of the barren or sparsely vegetated is higher than the other three underlying surface types, while the average LST of snow and ice is the lowest. Figure 10b demonstrates the diurnal variations of the LST of those different underlying surfaces. The LST diurnal variation of four underlying surfaces also show single peak curve and the maximum value occurred at 2:00 p.m. BST. Figure 10c shows the average LST of four underlying surfaces in summer and winter. The annual LST range of four different land cover types were 33.45 K (barren or sparsely vegetated), 8.74 K (snow and ice), 27.02 K (grasslands) and 9.06 K (water), respectively. Figure 10d demonstrates the diurnal LST range of four different underlying surfaces. The diurnal variation of LST is the largest at the barren or sparsely vegetated (22.83 K), second at the grasslands (19.82 K), third at the snow and ice (5.45 K) and smallest at the water (5.12 K). The above results can be explained by the different thermodynamic properties of the four underlying surfaces. The greater the thermal capacity and the thermal inertia of the underlying surface, the smaller the diurnal and annual range of the LST. This indicates that LST is closely connected with land cover types. The thermodynamic properties of the underlying surface play key parts in the diurnal variation and seasonal variation of the LST.

## 5. Conclusions

Due to the complex terrain and complicated weather conditions of the TP, the field observation is rather difficult. For the time being, no time series of LST with high temporal resolution is available over the TP. This greatly limits the study on the energy and water cycle over this specific region. On the other hand, the SWAs have been widely used to retrieve LST from the remote sensing point of view. Even though more than 20 SWAs have been published, actually no universal SWA can be applied everywhere, especially for the TP area with heterogeneous land surface status. In this study, the BL95 SWA was found to have some large discrepancies over the TP. Since we have some unique observation data, with the aid of accurate WVC data, the coefficients in BL95 were improved to be suitable for the TP. The coefficients in BL95 were derived on a monthly basis. Compared with the previous method, the modified SWA has been validated with reasonable higher accuracy, which is also proved to be superior to the MODIS LST product. Therefore, in this way, the BL95 algorithm has been extended to the TP area with complex terrain. The main conclusions are as follows.

(1) A comparison was carried out between two WVC reanalysis products and in situ GPS data of the JICA project. Except MB (−0.157 $g/{\mathrm{cm}}^{2}$) and R (0.836), the NCEP CFSR product shows lower RMSE (0.295 $g/{\mathrm{cm}}^{2}$) and lower MAE (0.230 $g/{\mathrm{cm}}^{2}$) than the MERRA-2 product (with RMSE, MB, MAE and R values of 0.301 $g/{\mathrm{cm}}^{2}$, −0.153 $g/{\mathrm{cm}}^{2}$, 0.232 $g/{\mathrm{cm}}^{2}$ and 0.841, respectively). The accuracy of NCEP CFSR WVC product is proved to be higher than that of MERRA-2 product over the TP area.
(2) An improved SWA was developed to retrieve the LST over the TP. The validation results show that the improved SWA could provide a better estimation of LST than the BL95. The LST retrieved through the improved SWA is closer to the in situ observations (with RMSE, MB, MAE, R values of 2.26 K, 0.83 K, 1.91 K, and 0.987, respectively), and its spatial patterns conform to the status of land surface well. The retrieval of the LST is also found to be superior to the MODIS LST product over the TP. The retrieval LST has a lower RMSE (3.49 K), MB (0.10 K) and MAE (2.58 K) and a higher R (0.937) than the MODIS product (with RMSE, MB, MAE and R values of 6.80 K, −2.10 K, 5.42 K, and 0.794, respectively).
(3) Through the MVC method, the time series data of the LST, daily maximum LST and diurnal variation of the LST are established over the TP. The results reveal the spatial and temporal distribution of the LST, daily maximum LST and diurnal variation of LST in the TP area in detail. The daily maximum LST was lower in the central part of the TP and higher in the southern and western parts of the TP and the Qaidam Basin area. Combined with results from previous research, it was found that the diurnal range of the LST over the TP could be affected by the summer monsoon evolution and the thawing and freezing of soil. The diurnal variation of the LST decreased when the soil became wetter because of the monsoon rain and soil thawing process.
(4) Four different typical underlying surface types were chosen to further study the effects of the different underlying surfaces on the LST. The results show that the LST is closely connected with land cover types. The thermodynamic properties of the underlying surface play key parts in the diurnal variation and seasonal variation of the LST. The greater the thermal capacity and the thermal inertia of the underlying surface, the smaller the diurnal and annual range of the LST.

It must be noted that there are some limitations of this study. Although the SWA has been widely used in the estimation of the LST, it is greatly influenced by the accuracy of the atmospheric WVC. According to the above sensitivity analysis, the improved SWA is greatly affected by the WVC accuracy. Meanwhile, the resampling of the WVC data might influence the precision of the LST retrievals. However, for the time being, it is also unavoidable because there is a lack of the high spatio-temporal resolution of WVC products over the TP. Moreover, the retrieval LST from the FY-2C is representative at the 5 km scale, while the in situ measurements can only represent a smaller scale that is focused around the observation stations. The scale difference of the instruments might cause some uncertainties. Finally, the temporal difference among in situ measurements, and the overpassing time of FY-2C and MODIS will lead to some discrepancies.

In the next step, combining the China Meteorological Forcing Dataset and other land surface characteristic parameters (NDVI, albedo, and emissivity) retrieved from the polar-orbiting satellite data, the time series of hourly LST can be considered as the input data for the Surface Energy Balance System (SEBS) model to derive the diurnal variations of the surface energy balance components over the TP. This will help to further understand the land–atmosphere interactions over the TP at a higher temporal resolution.

## Figures and Tables

**Table 1:** The specifications of FY-2C/SVISSR channels.

Channel No.	Channel Name	Spectral Range (μm)	Spatial Resolution (km)
1	FIR1	10.3–11.3	5
2	FIR2	11.5–12.5	5
3	FIR1	6.3–7.6	5
4	MIR	3.5–4.0	5
5	VIS	0.55–0.90	1.25

**Table 2:** Ground observation stations.

Sites	Longitude (°E)	Latitude (°N)	Altitude (m)	Land Cover
✰ BJ	91.89871	31.36859	4509.0	alpine and subalpine meadow
✰ D66	93.78454	35.52353	4585.0	alpine and subalpine plain grass
✰ D105	91.94256	33.06429	5039.0	alpine and subalpine plain grass
✪ Linzhi	94.73840	29.76450	3326.0	needle-leaved evergreen forest
✰ Nam Co	90.98850	30.77500	4730.0	alpine and subalpine meadow
✰ QOMS	86.94640	28.35810	4276.0	alpine and subalpine plain grass
✰ MS3478	91.71600	31.92600	4620.0	alpine and subalpine meadow
✰ MS3608	91.78328	31.22623	4588.9	alpine and subalpine meadow
◯ GAIZ	84.06221	32.30626	4394.3	alpine and subalpine plain grass
◯ GANZ	99.99755	31.61966	3357.8	alpine and subalpine meadow
◯ LITA	100.27077	29.99468	3925.2	alpine and subalpine meadow
◯ LNGZ	92.46006	28.41416	3824.4	alpine and sulpine meadow
◯ NAQU	92.06118	31.47977	4477.8	alpine and subalpine plain grass
◯ RUOE	102.96581	33.57598	3417.8	alpine and subalpine meadow
◯ SHEN	88.70490	30.93161	4635.9	alpine and subalpine plain grass
◯ DEQN	98.90737	28.48851	3295.0	needle-leaved evergreen forest
◯ DING	95.59356	31.41513	3843.0	alpine and subalpine meadow
◯ DINR	87.12039	28.65461	4326.6	alpine and subalpine plain grass
✰ RanwuM	96.7711	29.4811	3928.0	water
✰ RanwuD	96.6477	96.6477	3923.0	water

✰ Meteorological stations, ◯ GPS stations, ✪ Meteorological stations and GPS stations.

**Table 3:** The statistics of two reanalysis WVC data versus GPS measurements at 11 stations.

Sites	NCEP CFSR				MERRA-2				N
	RMSE	MB	MAE	R	RMSE	MB	MAE	R	
	($g/{\mathrm{cm}}^{2}$)	($g/{\mathrm{cm}}^{2}$)	($g/{\mathrm{cm}}^{2}$)		($g/{\mathrm{cm}}^{2}$)	($g/{\mathrm{cm}}^{2}$)	($g/{\mathrm{cm}}^{2}$)		
GAIZ	0.224	−0.074	0.171	0.901	0.230	−0.104	0.176	0.906	1892
GANZ	0.445	−0.336	0.362	0.925	0.455	−0.354	0.371	0.931	2457
LITA	0.249	−0.102	0.193	0.918	0.210	−0.040	0.161	0.933	2512
LNGZ	0.351	−0.235	0.271	0.927	0.263	−0.117	0.200	0.919	1283
NAQU	0.200	−0.051	0.150	0.915	0.182	−0.037	0.135	0.928	2345
RUOE	0.249	0.031	0.189	0.891	0.285	0.046	0.206	0.872	2408
SHEN	0.279	−0.167	0.221	0.795	0.218	−0.051	0.173	0.821	1100
DEQN	0.280	−0.122	0.218	0.931	0.346	−0.241	0.278	0.930	1744
DING	0.212	−0.122	0.143	0.136	0.331	−0.183	0.186	0.146	38
DINR	0.301	−0.182	0.235	0.906	0.249	−0.131	0.187	0.915	1518
Linzhi	0.451	−0.364	0.376	0.955	0.545	−0.473	0.477	0.951	1675
**Mean**	0.295	−0.157	0.230	0.836	0.301	−0.153	0.232	0.841	

**Table 4:** FY-2C cloud classification data contents.

Data	Meaning
0	Clear Oceans
1	Clear Lands
11	Mixed Pixels
12	Altostratus or Nimbostratus
13	Cirrostratus
14	Cirrus Dens
15	Cumulonimbus
21	Stratocumulus or Altocumulus

**Table 5:** Split window algorithm coefficients ($a_{0}$–$a_{12}$) in Equation (5).

	Month												
	1	2	3	4	5	6	7	8	9	10	11	12	Water
$a_{0}$	20.76	−18.50	7.230	1.703	−19.70	28.28	48.61	183.33	37.73	76.23	−9.66	13.38	264.0
$a_{1}$	161.0	110.4	13.24	−3.36	22.86	11.08	17.42	5.56	39.92	−29.77	26.58	11.20	14.10
$a_{2}$	0.83	1.03	0.93	1.01	1.01	0.86	0.73	0.25	0.75	0.77	1.03	0.95	0.005
$a_{3}$	6.56	2.64	4.05	1.33	4.69	3.21	5.74	6.35	6.72	−1.72	−0.27	−0.30	4.12
$a_{4}$	−39.49	−24.08	−0.40	4.39	−3.24	−0.54	−2.33	−0.37	−8.47	10.37	−1.72	2.47	−3.87
$a_{5}$	0.49	0.89	−1.60	−0.17	−1.15	2.57	4.48	1.41	−4.00	1.87	0.09	1.73	6.09
$a_{6}$	−3.94	−4.93	1.74	−0.52	2.80	−1.77	−2.38	2.26	6.25	−0.93	−4.06	1.50	−4.19
$a_{7}$	−19.26	3.23	−3.66	12.29	4.39	−3.40	−4.20	16.63	3.86	4.63	−2.94	−0.26	90.86
$a_{8}$	144.19	75.65	24.08	−0.50	−18.24	7.98	−1.08	−23.19	14.73	6.88	60.95	92.00	−67.35
$a_{9}$	1408.72	−57.91	737.08	−184.39	−192.27	527.82	143.06	−684.25	97.52	−173.52	−198.25	−162.64	−5728.65
$a_{10}$	−8240.61	−4513.60	−1847.9	−915.76	888.10	−716.14	74.38	1242.25	−1145.41	−425.31	−2382.34	−3952.42	4281.47
$a_{11}$	−487.51	2.91	−748.48	−666.49	−102.93	282.94	1306.60	491.06	−810.21	103.19	167.09	12.23	−4781.65
$a_{12}$	1591.99	−1634.86	26.93	1280.16	476.18	−183.94	−378.13	−707.88	788.61	−83.32	−3452.47	730.95	3107.35

**Table 6:** Effects of WVC error on accuracy of retrieved LST.

Δw ($g/{\mathrm{cm}}^{2}$)	RMSE (K)	MB (K)	MAE (K)
0.40	6.30	5.02	5.39
0.30	4.72	3.77	4.04
0.20	3.14	2.51	2.69
0.10	1.57	1.26	1.35
−0.10	1.57	−1.26	1.35
−0.20	3.14	−2.51	2.69
−0.30	4.72	−3.77	4.04
−0.40	6.30	−5.02	5.39
